# Development of ^68^Ga-Labeled Hepatitis E Virus Nanoparticles for Targeted Drug Delivery and Diagnostics with PET

**DOI:** 10.1021/acs.molpharmaceut.2c00359

**Published:** 2022-07-20

**Authors:** Elisavet Lambidis, Chun-Chieh Chen, Mo Baikoghli, Surachet Imlimthan, You Cheng Khng, Mirkka Sarparanta, R. Holland Cheng, Anu J. Airaksinen

**Affiliations:** † Department of Chemistry, Radiochemistry, University of Helsinki, Helsinki FI-00014, Finland; ‡ Department of Molecular and Cellular Biology, University of California, Davis, California 95616, United States; § Turku PET Centre, Department of Chemistry, University of Turku, Turku FI-20520, Finland

**Keywords:** gallium-68, DOTA, positron emission tomography tracers, virus-like particle, hepatitis E viral nanoparticles, hepatotropism

## Abstract

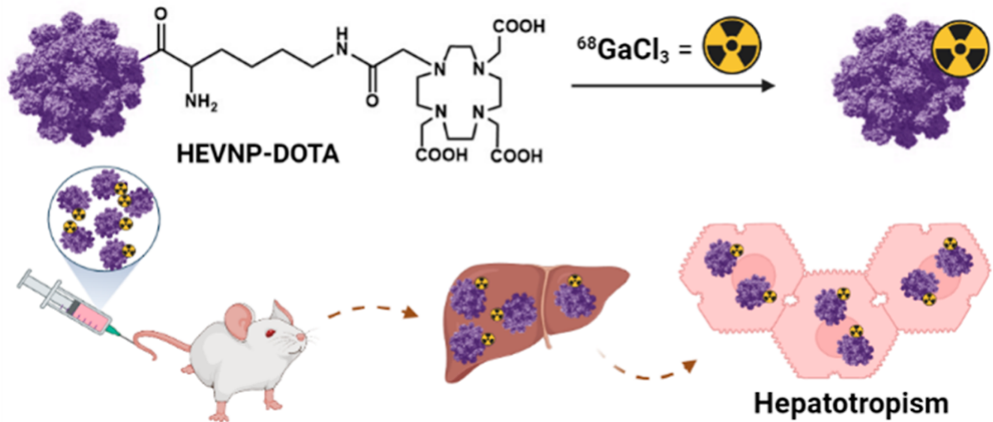

Targeted delivery of diagnostics and therapeutics offers essential advantages over nontargeted systemic delivery. These include the reduction of toxicity, the ability to reach sites beyond biological barriers, and the delivery of higher cargo concentrations to diseased sites. Virus-like particles (VLPs) can efficiently be used for targeted delivery purposes. VLPs are derived from the coat proteins of viral capsids. They are self-assembled, biodegradable, and homogeneously distributed. In this study, hepatitis E virus (HEV) VLP derivatives, hepatitis E virus nanoparticles (HEVNPs), were radiolabeled with gallium-68, and consequently, the biodistribution of the labeled [^68^Ga]Ga-DOTA-HEVNPs was studied in mice. The results indicated that [^68^Ga]Ga-DOTA-HEVNPs can be considered as promising theranostic nanocarriers, especially for hepatocyte-targeting therapies.

## Introduction

Virus-like particles (VLPs) are coat proteins fabricated from viral capsids and have been extensively investigated as potential drug delivery agents.^1−4^ For instance, various types of VLPs have been applied for targeted therapy utilizing receptors recognized by the VLPs.^5^ Examples are the canine parvovirus VLPs which recognize the transferrin receptors on tumor cells;^6^ rotavirus VLPs for intestinal cell uptake;^5,6^ and enterobacteria-phage VLPs, such as Qβ and MS2, for kidney, ovarian, and leukemia T cells.^7^ When a single system can combine the delivery and release of therapeutics with the ability to monitor the biodistribution and intracellular fate, it is called a theranostic system. Plant-derived VLPs, such as cowpea mosaic virus (CPMV) and cowpea chlorotic mottle virus (CCMV), have been studied in theranostic applications, such as imaging of the tumor endothelium (CPMV).^8,9^ Furthermore, the ordered and repeated surface of the supramolecular structure and the small size in the range of 20–100 d.nm (nm in diameter) of VLPs enable them to serve as vaccines.^8^ VLPs have been extensively used for this purpose as their vaccines are safer compared to viral vaccines.^10−15^

Hepatitis E virus nanoparticles (HEVNPs) are derived from a modified form of the HEV nonenveloped capsid protein (ORF2; 660 amino acids).^4,6,16−20^ HEVNPs are produced by the baculovirus expression system in insect cells,^1,2,4,6^ and they exhibit the physical characteristics of the authentic, low-virulence pathogen virus HEV while lacking the viral genome (RNA).^21^ As a consequence, they cannot replicate and are noninfectious and unable to generate virulence in patients.^6,22,23^ Furthermore, the recombinant HEVNPs are small (∼26 d.nm), biodegradable (into amino acids), highly biocompatible, and nontoxic.^24^ The HEVNP icosahedral capsid protein is highly ordered and symmetrical, allowing high stability toward chemical modifications.^6,24−27^ An important characteristic of HEVNPs is that the epitope disruption at the antibody-binding site of the engineered HEVNPs enables them to eliminate (or significantly reduce) their recognition from the HEV antibodies.^25,28^ Additionally, the capsid has the capability to be reversibly disassembled and reassembled through chemical reduction and chelation, providing a method for payload encapsulation to construct theranostic nanomaterials *in vitro*. The encapsulation into HEVNPs via an electrostatic interaction is feasible with, for example, negatively charged nucleic acids,^1^ nano-sized proteins,^29^ or inorganic NPs.^30^ Furthermore, HEVNP functionalization has been utilized to construct multifunctional systems, adjustable for noninvasive therapy, imaging, tissue-targeting,^25^ and vaccination^16,17^ and even for tumor-directed hyperthermia treatment induced by radiofrequency electromagnetic radiation.^2^

Highly interesting is the function of HEVNPs as liver-specific nanocarriers. Liver accumulation is a typical feature of nanomaterials resulting from their recognition by the liver-resident macrophages like Kupffer cells, leading to phagocyte-mediated clearance of the nanomaterials from the circulation.^7,31−33^ Nevertheless, unlike many other nanomaterials, HEVNPs have been demonstrated to inherently internalize into hepatocytes via receptor-mediated endocytosis, which is in agreement with the inherent liver tropism of the HEV itself.^19,34−37^

Heparan sulfate proteoglycans (HSPGs) and heat shock protein 90 (HSP90) are responsible cell proteins that allow HEV’s initial binding to the membrane and intracellular transportation in the liver cells, respectively.^12,35,36,38^ The exact mechanism was proven to be followed by HEVNPs.^27,35^ Furthermore, the entry of HEVNPs to hepatoma cells, such as Huh7 and SK-Hep-1, is achieved via dynamin-dependent and clathrin-mediated endocytosis (by budding into dynamin- or clathrin-coated pits leading to the internalization of nonenveloped particles in the form of vesicles).^12,27,35,37,39^ The entry of HEVNPs to hepatocytes can be blocked by inhibitors of clathrin-mediated endocytosis, confirming this as the pathway of endocytosis.^35,40^ Other VLPs might follow the internalization via clathrin-mediated endocytosis only if they are functionalized, for example, with transferrin for kidney entry.^7^ Overall, the uptake of HEVNPs has been characterized as liver-specific and serves as an excellent advantage for the delivery of various payloads to hepatocytes.^27,35^

Positron emission tomography (PET) is a widely used molecular imaging modality that enables noninvasive evaluation of PET agents, including theranostics, in terms of their biodistribution, targeting efficacy, and pharmacokinetics. In contrast with other imaging techniques, such as magnetic resonance imaging, PET is highly sensitive (up to picomolar concentration with subpharmacological doses) and enables quantitative analysis of the administered tracer distribution. The latter property characterizes PET as a functional imaging modality and reduces the required number of preclinical experiments profoundly and thus the number of animals. Additionally, unlike in optical imaging, limited tissue penetration of the signal is not an issue with PET.^41^

Gallium-68 is one of the most widely used radiometals for clinical PET diagnostics. Its physical half-life of 67.7 min is long enough to track the tracer for up to 2 h.^42^ One advantage of gallium-68 is its production from a portable and readily available ^68^Ge/^68^Ga generator. Gallium-68 is a hard Lewis acid and consequently binds strongly to hard Lewis bases, such as carboxylates and phosphonates. It preferably couples to strong donor ligands or bifunctional chelators (BFCs) such as 1,4,7,10-tetraazacyclododecane-1,4,7,10-tetraacetic acid (DOTA)-based BFCs. DOTA enables fast and efficient labeling with gallium-68 as well as good *in vivo* stability. Very well-known examples include the somatostatin receptor ligands [^68^Ga]Ga-DOTA-TOC, [^68^Ga]Ga-DOTA-NOC, and [^68^Ga]Ga-DOTA-TATE.^43,44^

Taking into consideration the numerous ideal characteristics of HEVNPs, this study aimed to develop a radiolabeling methodology for the efficient ^68^Ga-labeling of HEVNPs and to prove the capability of the particular nanosystem to serve as a potential new theranostic agent using PET. The biodistribution of [^68^Ga]Ga-DOTA-HEVNPs was evaluated both ex vivo after intravenous administration in Balb/c mice and *in vitro* in various cell lines, such as hepatocytes.

## Materials and Methods

All chemicals and solvents were obtained from commercial providers, and they were used without further purification. HEVNPs (in 10 mM 2-(*N*-morpholino)ethanesulfonic acid (MES) buffer, pH 6.2, >10 mg/mL) were prepared as previously described.^25^ 1,4,7,10-Tetraazacyclododecane-1,4,7,10-tetraacetic acid mono-*N*-hydroxysuccinimide ester (DOTA-NHS ester) was purchased from Macrocyclics (Plano, TX, USA). All water used was ultrapure (>18.2 MΩ cm^–1^) and was prepared on a Milli-Q (MQ) Integral 10 water purification system. For each buffer preparation, MQ water was treated with Chelex 100 sodium form (Sigma-Aldrich, St. Louis, MO, USA) at a concentration of 5 g/L for the elimination of trace metals. The PD-10 Sephadex G-25 M desalting columns were obtained from GE Healthcare (Chicago, IL, USA) and preconditioned prior to use with 20 mL of MQ water and 20 mL of phosphate buffer saline (PBS; 0.01 M, pH 7.4). The HEVNP size and morphology were determined by transmission electron microscopy (TEM) (JEOL1400, JEOL Ltd., Akishima, Tokyo, Japan). The zeta (ζ) potential was calculated from the electrophoretic mobility, and its distribution was measured in MQ using a ZetaSizer Nano instrument (Malvern Ltd.; Worcestershire, UK). The protein concentration was measured using a μDrop Plate on a Multiskan Sky Microplate spectrophotometer from Thermo Scientific (Waltham, MA, USA). For the ^68^Ga-elution, the water Tracer SELECT was acquired from Honeywell-Riedel-de Häen (Seelze, Germany), and the ultrapure 30% HCl (hydrochloric acid) was purchased from Merck (Kenilworth, NJ, USA). The ^68^Ge/^68^Ga generators (1.85 or 2.41 GBq at calibration, respectively) were GalliaPharm-type generators produced by Eckert & Ziegler (Berlin, Germany). The radiochemical purity was determined by Whatman 1 paper chromatography with 0.5 mM diethylenetriamine pentaacetate (DTPA) as a mobile phase. A photostimulated luminescence scanner FLA 5100 (Fujifilm, Tokyo, Japan) was used for the digital autoradiography using a Fuji TR323309 imaging plate and a 24 × 30 X-ray cassette. The automatic gamma counter was 1480 Wallac Wizard 3” (PerkinElmer Life Sciences, Waltham, MA, USA), and the measurement lasted for 60 s per tissue sample. The human hepatocyte carcinoma (Hep G2; HB-8065), colorectal carcinoma (HCT 116; CCL-247), and murine macrophage (RAW 264.7; TIB-71) cell lines were purchased from ATCC (Manassas, VA, USA). More information on cell culture conditions can be found in the Supporting Information.

### TEM Measurements

HEVNPs were negatively stained with 2% uranyl acetate (UA) of neutral pH and examined under a transmission electron microscope at various magnifications. The TEM grids were Cu 200 mesh normal bars with a carbon sputter coating. The exposure time of the NPs on the grid was 1 min, and the amount was 3 μL (PBS/MQ 1:10). Immediately after the removal of the excess solution, the staining of the NP on the grid was done by spotting 3 μL (always the same amount with the sample) of the UA solution on the carbon grid for 20 s. To analyze the TEM images of the NPs, Fiji ImageJ 1.51 software was used.

### Functionalization of the HEVNPs with DOTA for Radiolabeling

HEVNPs (1 mg, 23.34 mg/mL, 10 mM MES, pH 6.2) were diluted in 0.01 M phosphate buffer (PB) (pH 7.4) to a final volume of 250 μL (2 mg/mL or 37.6 μM, 18.8 nmoL). The DOTA-NHS ester (18 mg, 23.6 μmoL) was dissolved in 0.01 M PB pH 7.4 to a final concentration of 45 mM and a final volume of 250 μL. The NHS ester solution was added *dropwise* to the reaction tube containing the HEVNPs. The mixture was immediately mixed well by careful pipetting. Sodium hydrogen carbonate (0.1 M, pH 9) was used for adjusting the pH to 7.4. The final reaction volume was 1100 μL. The reaction mixture was shaken (500 rpm) at room temperature (RT) for 3 h and then at 4 °C overnight. A PD-10 column was used to purify the conjugated NPs by gravity size exclusion chromatography (SEC) by eluting the conjugated NPs with 3 mL of 0.01 M PBS (pH 7.4).

### Radiolabeling and Purification of the DOTA-Functionalized HEVNPs

DOTA-HEVNPs (0.13 mg/mL in 100 μL in PB) were diluted with metal-free 0.25 M ammonium acetate buffer (800 μL). This was followed by the addition of freshly eluted [^68^Ga]GaCl_3_ in 0.1 M HCl (10–79 MBq in 1 mL). The final reaction volume was 1.9 mL, and the reaction pH was 4.5. The reaction was mixed for 30 min at 61 ± 1.5 °C at 350 rpm. After 30 min, the reaction mixture was cooled down to RT and immediately purified using a preconditioned PD-10 column. The ^68^Ga-labeled HEVNPs were eluted with 2.8 mL of sterile 0.01 M PBS (pH 7.4). The radiochemical purity of the ^68^Ga-labeled HEVNPs was confirmed by radio-thin layer chromatography (TLC) (Figure S1 in the Supporting Information) and was >98%.

### *In Vitro* Stability of [^68^Ga]Ga-DOTA-HEVNPs

The ^68^Ga-labeled HEVNPs (700 μL in phosphate buffer) were added into Protein LoBind 1.5 mL microtubes (Eppendorf) containing 700 μL of PBS (0.01 M, pH 7.4), 100% human plasma, iron (FeCl_3_·6H_2_O, final concentration: 18 mM in PBS), or a CO_2_-independent cell medium. All the samples (n = 3 per testing solution) were incubated at 37 °C under constant shaking. The amount of the intact radiolabeled HEVNPs was monitored by radio-TLC up to 6 h and starting from the first 10 min of incubation.

### *Ex Vivo* Biodistribution of [^68^Ga]Ga-DOTA-HEVNPs

All animal experiments were carried out under a project license approved by the National Board of Animal Experimentation in Finland (ESAVI/12132/04.10.07/2017) and in compliance with the respective institutional, national, and EU regulations and guidelines. The mice were group-housed in standard polycarbonate cages with aspen bedding, a nesting material (Tapvei, Harjumaa, Estonia), and enrichment (aspen blocks and disposable cardboard hut). Pelleted food (Teklad 2019C diet, Envigo, Horst, Netherlands) and tap water were available ad libitum. Environmental conditions of a 12:12 light/dark cycle, a temperature of 22 ± 1 °C, and a relative humidity of 55 ± 15% were maintained throughout the study.

The *ex vivo* biodistribution was studied in 20 healthy female BALB/c mice (Janvier Laboratories (France), 7–8 weeks, 16–21 g, 5 animals/time point). [^68^Ga]Ga-DOTA-HEVNPs (0.047 mg/mL, 0.2–0.5 MBq) were injected into the tail vein in 130–200 μL of PB pH = 7.4. The mice were sacrificed with CO_2_ asphyxiation 15, 30, 60, and 120 min after the injection, and selected organs were harvested. The harvested organs were weighed and counted by a gamma counter, and the radioactivity decay was corrected to the start of the measurement. Standards (five) were prepared using 10 μl of the formulated [^68^Ga]Ga-DOTA-HEVNP suspension and measured before the tissues. Results are expressed as percent of injected dose per gram of tissue (%ID/g).

### Cell Studies of [^68^Ga]Ga-DOTA-HEVNPs and Control in Hepatocytes, Macrophages, and Colorectal Cancer Cells

The day before the experiment, RAW 264.7 (macrophages), Hep G2 (hepatocytes), or HCT 116 (colorectal cancer) cells were plated on sterile 6-well plates (50,000 cells/mL/well or 2.2 million HCT 116 cells/mL/well). The following day, the cells were incubated at 37 °C with either [^68^Ga]Ga-DOTA-HEVNPs or the free ^68^Ga-radionuclide diluted in 1 mL of fresh culture medium (HEVNPs, 3 μg, 0.2 MBq/mL/well and [^68^Ga]GaCl_3_, 0.01 MBq/mL/well). The incubation time varied depending on the time point (15, 30, 60, and 120 min). The detailed cell uptake procedure is given in the Supporting Information.

### Statistical Analysis

The average values of the quantitative data were analyzed for statistical significance using an unpaired two-tailed *t*-test on GraphPad Prism 9 (San Diego, CA, USA), with a *P* value equal or less than 0.05 considered as statistically significant [*P ≤ 0.05, **P ≤ 0.01,***P ≤ 0.001, ****P ≤ 0.0001 and nonsignificant (ns) when *P* > 0.05].

## Results and Discussion

**Scheme 1:**
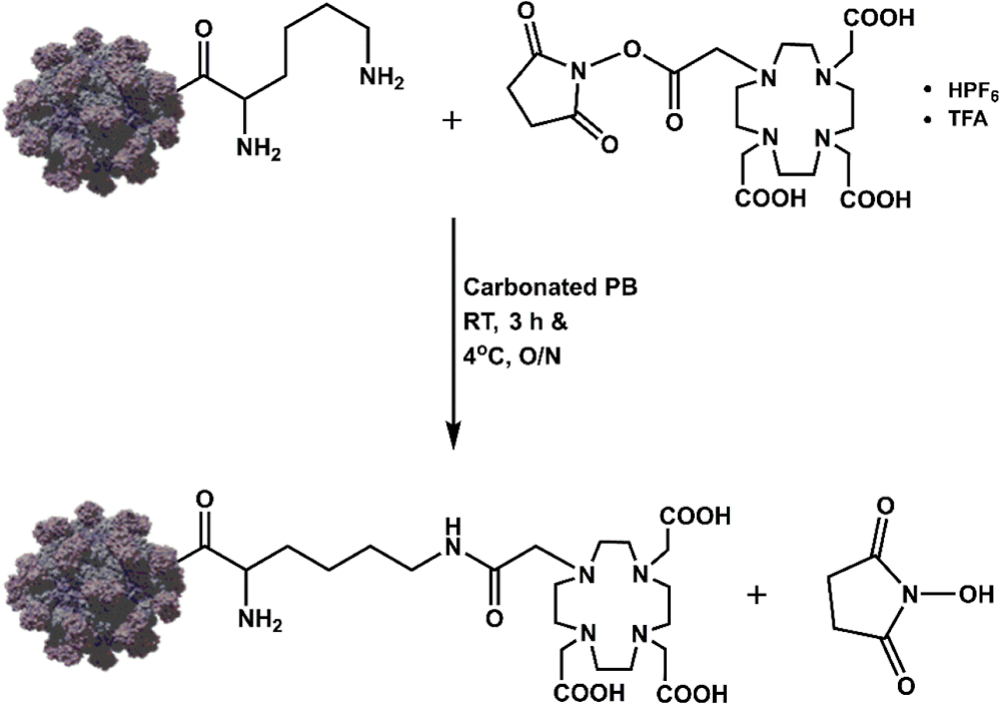
Synthesis of DOTA-HEVNPs Conjugation reaction between the NHS ester and the lysine primary amines on the HEVNP surface in PB at pH 7.4. The reaction was run overnight at 4 °C. RT = room temperature, O/N = overnight, HPF_6_ = hexafluorophosphoric acid, and TFA = trifluoroacetic acid.

In order to radiolabel HEVNPs, their surface was first functionalized with an appropriate chelating agent for gallium-68 (Scheme 1). In the specific study, this was achieved by the direct conjugation with the DOTA-NHS ester. Among the various amino acids that are present on the exterior of the HEVNPs, more specifically on the protrusion domain, amino acids cysteine and lysine exist in high quantities (*n* = 60 of each of the amino acids per HEVNP).^45^ Here, the primary amine of surface-exposed lysine residues was reacted with the NHS ester molecule to form an amide covalent bond. The amide bond formation with NHS is typically carried out in alkaline pH 8–9. However, this is not recommended for HEVNPs due to instability concerns at high pH.

**Figure 1:**
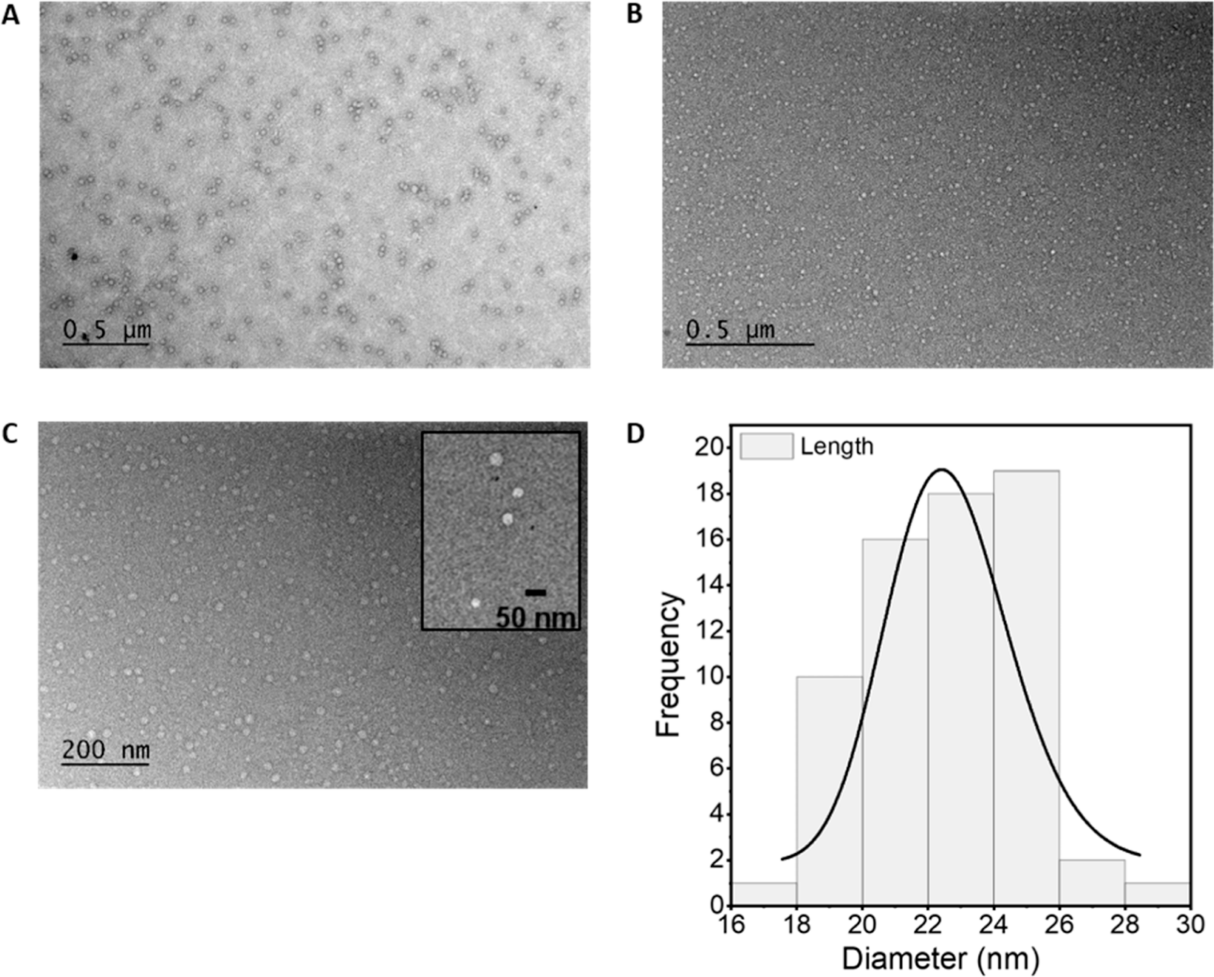
The size and morphology of the HEVNPs were characterized by TEM. (A) Unmodified spherical 20–30 d.nm HEVNPs in PBS/MQ 1:10. (B,C) HEVNPs after the PD-10 purification of the DOTA-conjugated HEVNPs (with a zoomed representation as an inset) in PB/MQ 1:10, 500 (B) and 200 nm (C) scale bars. The retention of the spherical shape of the NPs after the conjugation and purification was verified. The histogram (D) also confirms that the size of the measured DOTA-HEVNPs was kept in the range of 20–30 d.nm. The TEM images were analyzed with Fiji ImageJ 1.51.

For this reason, the reaction was done overnight in physiological pH, followed by purification of the DOTA-conjugated HEVNPs with SEC. The morphology and size of the DOTA-HEVNPs were examined using TEM (Figure 1). The intact spherical shape of HEVNPs of size within the expected range of 20–30 d.nm per NP was confirmed, and the appearance of the conjugated NPs in TEM corresponded well with that of the unconjugated HEVNPs. Additionally, no aggregation, and thus no NP cross-linking, was detected after the conjugation and purification steps. The ζ-potential of the NPs before and after the conjugation was also measured in order to ensure that the HEVNP surface and its potential remained unchanged after the modification. It was important that the degree of labeling would not affect the HEVNP properties and biodistribution with respect to the native NPs. The ζ-potential values were −22.03 mV for the stock HEVNPs and −21.7 mV for DOTA-HEVNPs, illustrating that the surface properties of the HEVNP were maintained.

The efficient conjugation of DOTA to the HEVNP was confirmed by the successful radiolabeling. [^68^Ga]Ga-DOTA-HEVNPs with a radiochemical yield of 64.2 ± 15.1% (*n* = 5) were purified with a PD-10 desalting column and were isolated with a high radiochemical purity of 98.6% ± 0.9 (*n* = 5). Additionally, the radiochemical yield from the radiolabeling of unmodified HEVNPs with [^68^Ga]GaCl_3_ was only 0.2% (*n* = 1), confirming the requirement of a chelating agent on the surface of the NPs for efficient radiolabeling. Moreover, this was a good indication that the trivalent ^68^Ga^3+^ does not get adsorbed on HEVNPs which would result in the quick release of free ^68^Ga^3+^ and nonspecific radiometal accumulation *in vivo*.

**Figure 2:**
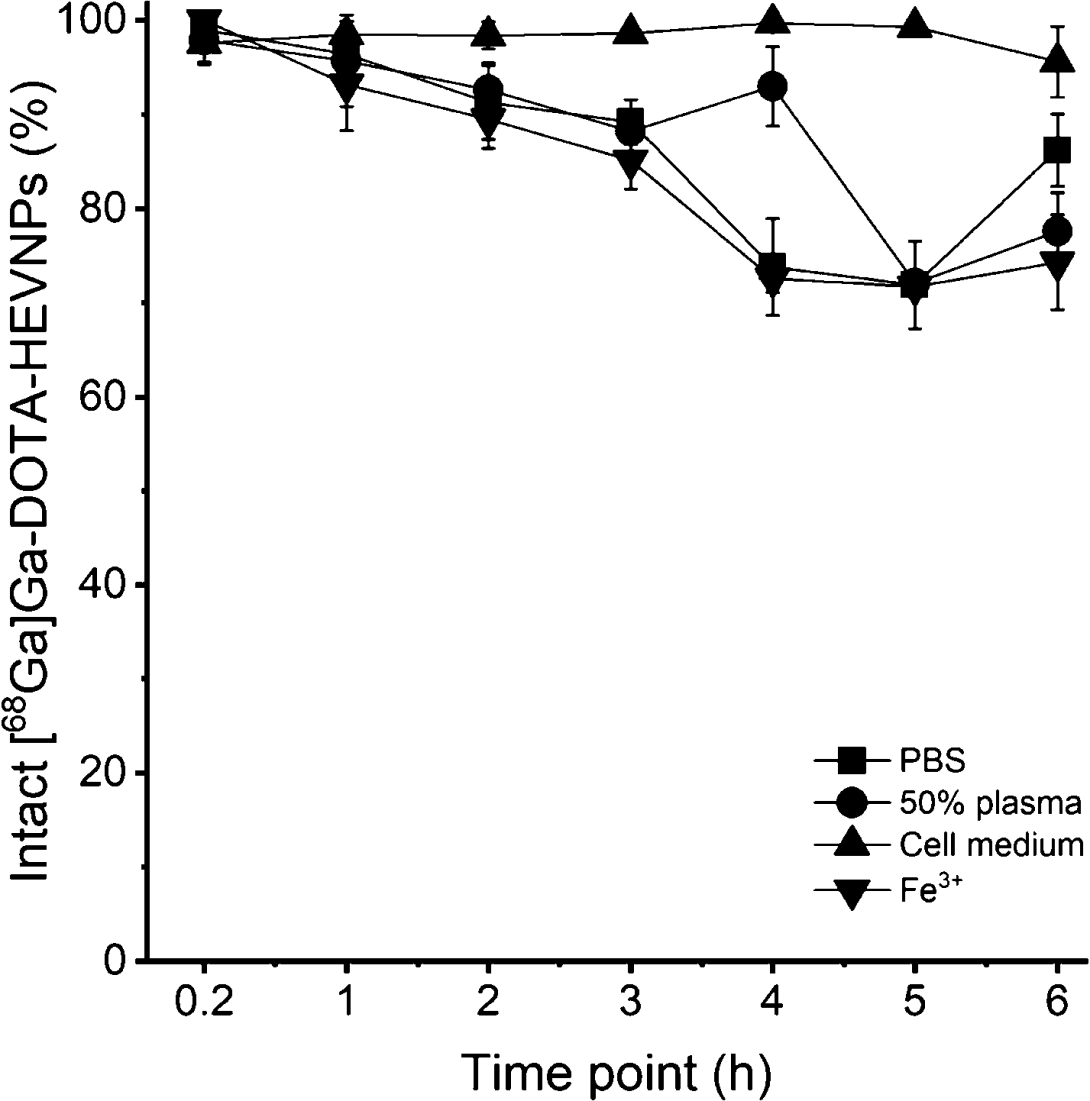
Stability assay of [^68^Ga]Ga-DOTA-HEVNPs at 37 °C under constant shaking. The labeled HEVNPs were tested in PBS (0.01 M, pH 7.4), 50% human plasma, CO_2_-independent cell culture medium, and iron (FeCl_3_·6H_2_O, 18 mM) for the iron challenge. Radio-TLC from the samples was done at seven time points up to 6 h in total. The values represent the average ± standard deviation (*n* = 2–3).

The stability of [^68^Ga]Ga-DOTA-HEVNPs was analyzed by an *in vitro* stability assay under four different conditions in PBS (pH = 7.4), 50% human plasma, and a cell culturing medium and with an iron challenge (Figure 2). Iron is an endogenous trace element and a trivalent cation. It can compete with ^68^Ga^3+^ in binding to the chelator *in vivo*. In addition, for the cell studies, the presence of free gallium-68 could affect the reliability of the outcome. For this reason, a high concentration of Fe^3+^ was used (18 mM) to count as an excess of the normal iron levels in the blood (maximum physiological amount in blood for males: 32 μM^46^). The stability of the radiolabel was investigated by radio-TLC (*R*_f_([^68^Ga]Ga-DOTA-HEVNPs) = 0.0 and *R*_f_([^68^Ga]GaCl_3_) = 0.8, Figure S1 in the Supporting Information). During the first time points, 10 min and 1 h, the radiolabel remained stable (>95.5%) in all test media with the exception at 1 h in Fe^3+^ (93.2 ± 4.9%). At the 2 h time point, the intact radiolabeled NPs were at least 90%, and the most profound loss of the radiolabel was detected from 4 h onward. However, due to the short physical half-life of gallium-68 (*t*_1/2_ = 67.7 min), the most important time points are the early ones (up to 1 h), in which [^68^Ga]Ga-DOTA-HEVNPs showed minimal radiolabel loss in a range of challenging conditions. Lastly, the cell medium used in subsequent assays was shown not to influence the HEVNP radiolabel stability.

Next, the *ex vivo* biodistribution of [^68^Ga]Ga-DOTA-HEVNPs was evaluated in BALB/c mice (total *n* = 20 mice, *n* = 5 per time point). [^68^Ga]Ga-DOTA-HEVNPs in 0.01 M PB (0.2–0.5 MBq in 200 μL per animal at the time of injection) were intravenously administered into the tail vein of awake mice.

**Figure 3:**
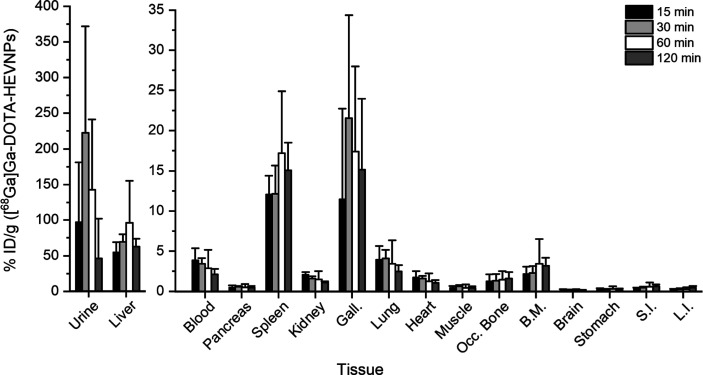
The *ex vivo* biodistribution of [^68^Ga]Ga-DOTA-HEVNPs after the i.v. injection in healthy BALB/c mice showed fast urinary elimination accompanied by high radioactivity levels in the liver. The columns represent the average ± standard deviation (*n* = 3–5). %ID/g = percent of injected dose per gram of tissue, Gall.: gallbladder, SI: small intestine, LI: large intestine, Occ. Bone: occipital bone, and BM: bone with marrow.

As can be seen in Figure 3 (and Table S1 in the Supporting Information), increased radioactivity was observed in the liver and spleen already at 15 min after the injection. The highest radioactivity values in both the liver and spleen were obtained at 1 h post-injection (96.3 ± 58.7 and 17.2 ± 7.7% ID/g, respectively). The detected radioactivity in the liver was much more prominent than it is typically observed for VLPs of comparable size. For example, ^125^I-labeled BK and JC VLPs were initially detected in the liver (48 and 60% of recovered radioactivity in 10 min, respectively), but the radioactivity dropped at 1 h after the injection (about 40 and 30% of recovered radioactivity, respectively). The drastic decrease of the liver-associated radioactivity of the labeled JC VLPs was due to the effective hepatocellular degradation of the JC VLPs by the liver-resident macrophages, the Kupffer cells.^47^ Another example is the ^68^Ga-labeled CPP-gVLPs (cell-penetrating peptide-modified green-fluorescent VLPs) which showed only minor liver uptake with 1.12 ± 0.02% ID/g at 1 h after intravenous injection, which increased to 1.18 ± 0.01% ID/g within 2 h.^48^ In contrast, the [^68^Ga]Ga-DOTA-HEVNPs in this study reached over 50% ID/g in the liver after 15 min, and this almost doubled at the 1 h time point, consistent with the expected liver tropism of HEVNPs.

In the spleen, the [^68^Ga]Ga-DOTA-HEVNP accumulation was less than would be typically expected for NPs.^49^ The size of [^68^Ga]Ga-DOTA-HEVNPs is below 30 nm, and therefore, the observed spleen accumulation was unlikely due to the capillary sequestration of the particles from the circulation. Macrophage recognition might have contributed to the splenic uptake instead.

Additionally, increased radioactivity was observed in the gallbladder with the highest values attained at 30 min after intravenous injection (21.5 ± 12.8% ID/g), indicating that some HEVNPs and/or their fragments could be degraded in the liver and eliminated via the hepatobiliary system. However, even higher radioactivity levels were observed in the urine starting from the early time points. These high radioactivity levels in the urine could be due to the HEVNP radiometabolites, fragments, or the HEVNPs themselves even if they are larger than the molecular weight or size cutoff for the glomerular filtration (6 nm in the diameter size of the NP^50^) due to their flexible nature.^41^

**Figure 4:**
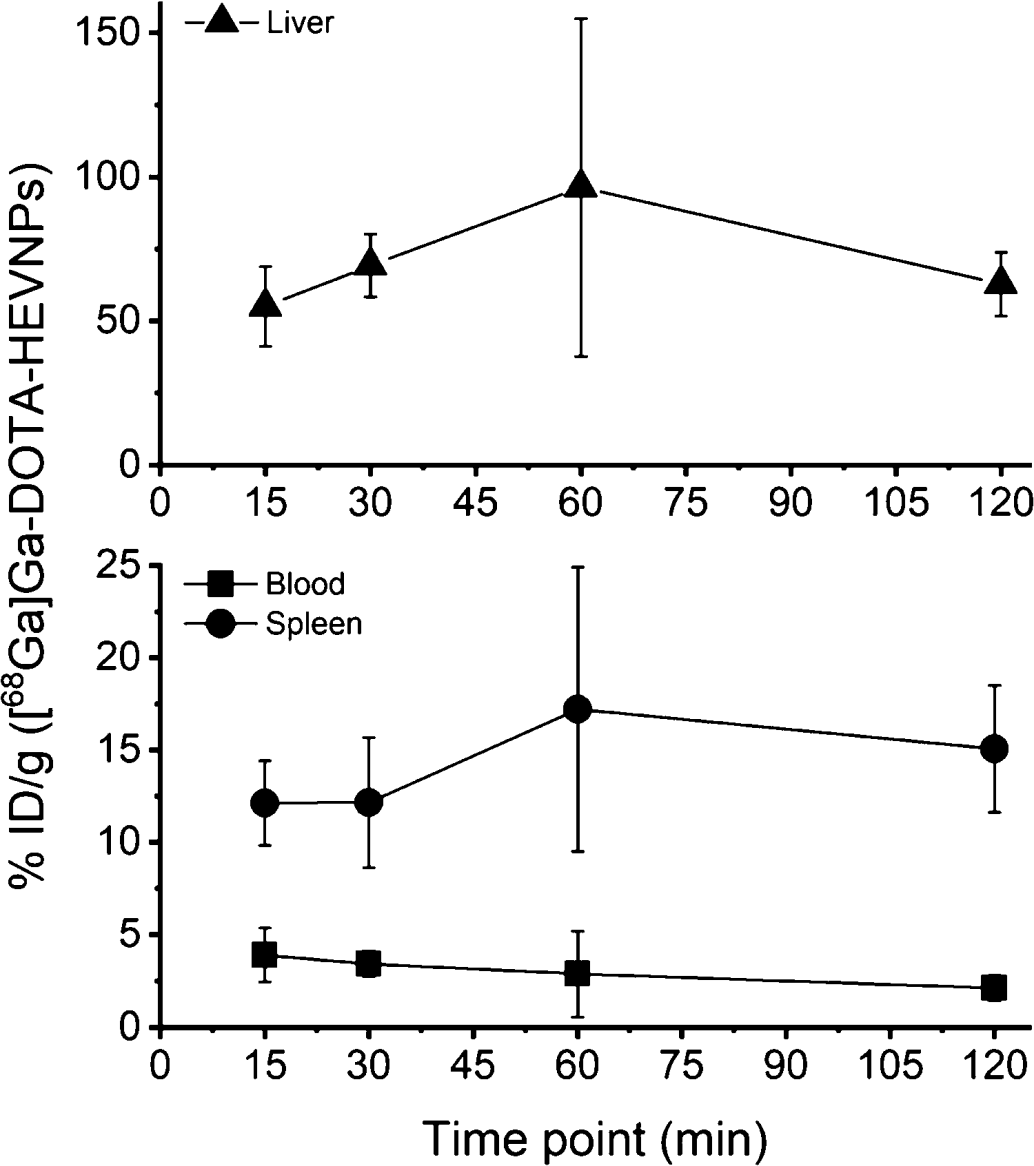
Activity level of [^68^Ga]Ga-DOTA-HEVNPs in the blood, spleen, and liver against time following the *ex vivo* biodistribution in healthy BALB/c mice. The values represent the average ± standard deviation (*n* = 3–5). % ID/g = percent of injected dose per gram of tissue.

**Figure 5:**
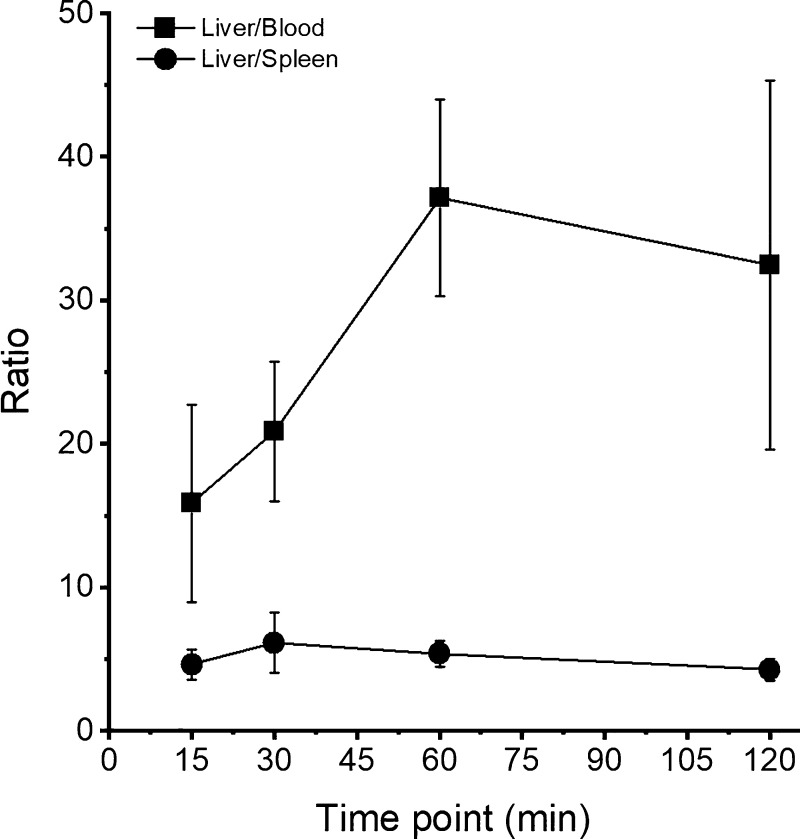
Comparison of the activity level (%ID/g) of [^68^Ga]Ga-DOTA-HEVNPs in the liver/blood and liver/spleen ratio against time following the *ex vivo* biodistribution in healthy BALB/c mice. The highest liver/blood ratio was attained at 1 h post-injection, whereas the liver/spleen ratio remained constant at 5 with a negligible increase at 30 min (6.14 ± 2.08). The values represent the average ± standard deviation (*n* = 3–5).

The activity levels in the blood, spleen, and liver and the ratio of liver-to-spleen and liver-to-blood are shown in separate graphs (Figures 4 and 5, respectively). Figure 4 shows the increased uptake of the labeled HEVNPs in the liver which is about 4 times higher than the uptake in the spleen. In addition, the blood retention of [^68^Ga]Ga-DOTA-HEVNPs was below 5% ID/g from the first 15 min post-injection. At the last time point (120 min), the NPs were still detected in the circulation (2.11 ± 0.67% ID/g). Moreover, the liver-to-blood ratio at 1 h post-injection was 37.11 ± 6.85, whereas the liver-to-spleen ratio was 5.38 ± 0.91 (Figure 5). Overall, the above results are in agreement with a previous study with fluorescently labeled HEVNPs in breast tumor-bearing BALB/c mice. Optical imaging revealed the accumulation of HEVNPs in the liver and spleen at 1 h post-injection time but did not allow quantification of organ uptake.^24,25^

**Figure 6:**
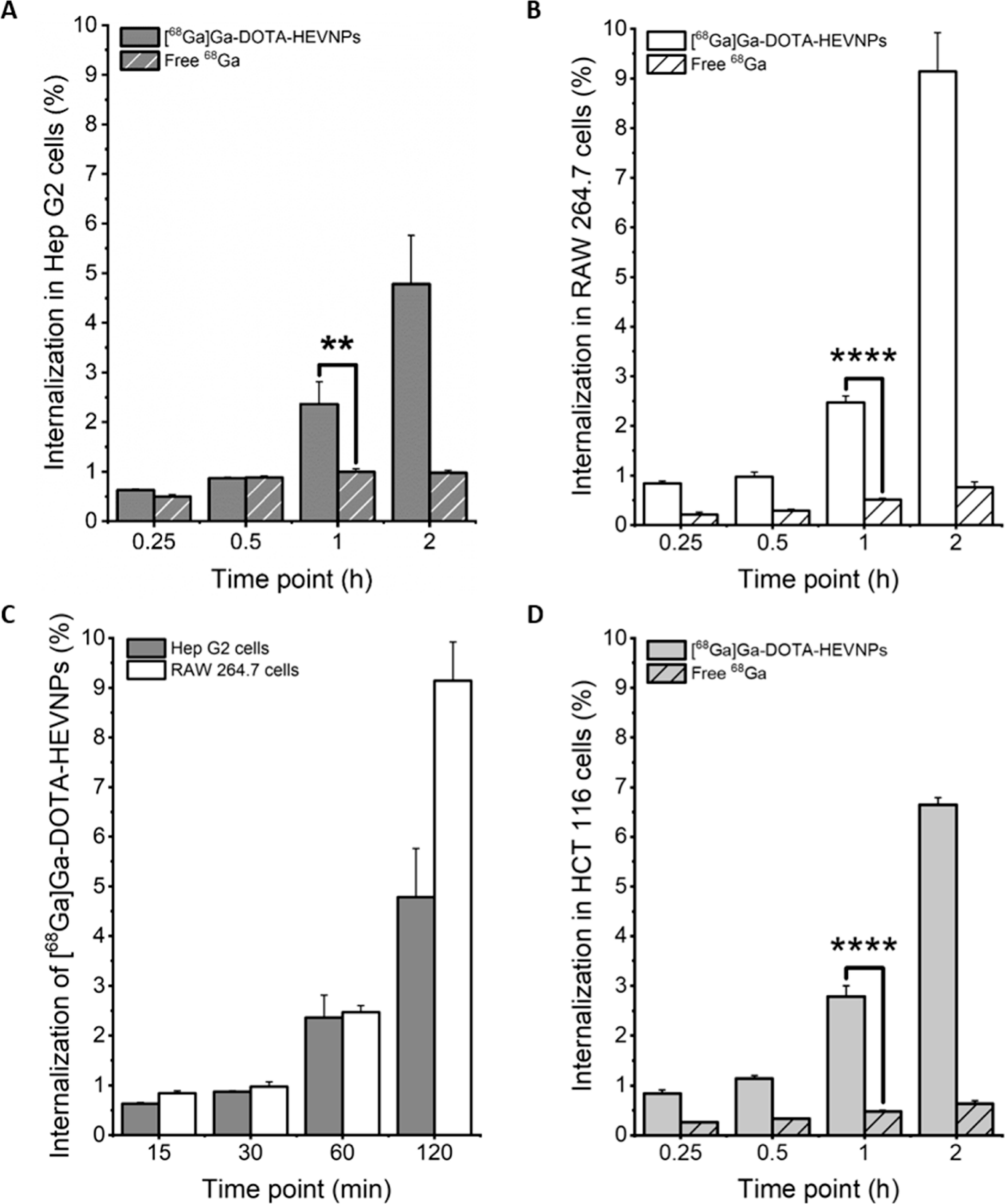
Cell internalization of [^68^Ga]Ga-DOTA-HEVNPs and [^68^Ga]GaCl_3_ in (A) hepatocytes (Hep G2) and (B) macrophages (RAW 264.7). The comparison between the two cell lines is shown for [^68^Ga]Ga-DOTA-HEVNPs (C). The cell uptake assay was done at 37 °C up to 2 h after the initiation of the incubation. The columns represent the average ± standard deviation (*n* = 3). An unpaired *t*-test was performed to assess the statistical significance of the difference between the HEVNPs and the free [^68^Ga]GaCl_3_ (Hep G2 (A): **P = 0.0063, RAW 264.7 (B) and HCT 116 (D): ****P < 0.0001 (D: N/A)).

Although from the results presented it could be assumed that the observed high liver uptake in our case was due to the liver specificity of the HEVNPs in general, we decided to further investigate this hypothesis prior to making any solid conclusions on the liver tropism of our HEVNP system. An *in vitro* study was conducted in order to compare the cell uptake of radiolabeled HEVNPs in macrophages and hepatocytes (Figures 6 and S2 in the Supporting Information). The two cell lines, RAW 264.7 murine macrophages and Hep G2 human hepatocytes, were treated with [^68^Ga]Ga-DOTA-HEVNPs at 37 °C. The time points were 15, 30, 60, and 120 min. In parallel, the cells were treated with [^68^Ga]GaCl_3_ as a negative control for cell internalization. The results revealed that [^68^Ga]Ga-DOTA-HEVNPs were internalized in both cell lines. Figure 6A–C shows that the internalization of [^68^Ga]Ga-DOTA-HEVNPs in macrophages and hepatocytes is approximately the same up to 1 h and with a maximum difference of 1.5% between the values for the two cell lines at the other time points. The NP internalization after 1 h of incubation was highly similar for the two cell lines with 2.36 ± 0.45% in Hep G2 and 2.47 ± 0.13% in RAW 264.7. For both cell types, a more profound increase in the uptake of [^68^Ga]Ga-DOTA-HEVNPs was observed from 1 to 2 h (2.4% difference in Hep G2 and 6.7% in RAW 264.7). By comparing the two cell lines, it can be seen that after 2 h of incubation, the NPs showed a higher degree of internalization in the RAW 264.7 cells which was double the amount detected in the Hep G2 cells at the same time point (9.14 ± 0.78 and 4.78 ± 0.98% in 2 h, respectively). Nevertheless, a noteworthy level of internalization for HEVNPs was also observed in the hepatocytes.

Clathrin-mediated endocytosis is one of the main mechanisms for HEV and HEVNP internalization into hepatocytes.^27,35,36,38^ Clathrin-mediated endocytosis is also an important mechanism of internalization in some cancer cell lines, for instance, in the HCT 116 colorectal cancer cells.^51^ It has been shown that lysine-rich molecules specifically interact with the cell membrane of the HCT 116 cells and get internalized via clathrin-mediated endocytosis.^52^ HEVNPs are lysine-rich and contain 60 lysine residues/particle. Therefore, the internalization of the HEVNP system was also examined in the HCT 116 colorectal cancer cells under the same conditions as it was done for the other two cell lines (Hep G2 and RAW 264.7). The cell uptake trend in the HCT 116 cells (Figure 6D) is very comparable to that in the Hep G2 and RAW 264.7 cells. Starting with <1% internalization in the first 15 min, [^68^Ga]Ga-DOTA-HEVNPs reached 2.78 ± 0.22% internalization in 1 h and a maximum of 6.64 ± 0.15% in 2 h, thus confirming the expected uptake in the HCT 116 cells. Lastly, it is worth noting that the internalization of the free radionuclide control was constant and in the range of 0.2–1% in all the cell lines throughout the study, confirming that the internalized radioactivity is due to the intact HEVNPs themselves (Figure 6A,B,D) and not the released radiolabel. This was further supported by the observed high *in vitro* radiolabel stability of [^68^Ga]Ga-DOTA-HEVNPs in the CO_2_-independent culture medium used in the assays.

Overall, it was confirmed *in vitro* that the developed [^68^Ga]Ga-DOTA-HEVNP system internalizes into hepatocytes from the first hour of incubation and follows a higher degree of internalization after 2 h. The liver cell uptake of [^68^Ga]Ga-DOTA-HEVNPs was expected with respect to the retained physical characteristics from the native HEV which display proven liver tropism due to clathrin-mediated endocytosis.^35,36,38^ Therefore, [^68^Ga]Ga-DOTA-HEVNPs could be described as promising natural viral capsid-based NPs for the targeted drug delivery and diagnostics of cancer, such as liver cancer, taking advantage of the hepatic specificity of HEVNPs.

## Conclusions

The HEVNP surface was functionalized with the DOTA bifunctional chelator using the exposed lysine residues on the surface of the NPs. The stability of HEVNP conformation was confirmed by TEM. DOTA-HEVNPs were then successfully radiolabeled with gallium-68 under mild reaction conditions. The high stability of the [^68^Ga]Ga-DOTA-HEVNPs was confirmed *in vitro* at 37 °C in physiological media and when challenged with Fe^3+^. The *ex vivo* biodistribution of [^68^Ga]Ga-DOTA-HEVNPs in mice revealed high liver uptake and low uptake in other organs. This was further investigated *in vitro* by comparing the HEVNP uptake in macrophages, hepatocytes, and colorectal carcinoma cells. To our knowledge, this is the first study reporting the radiolabeling of HEVNPs with a PET-compliant radionuclide and quantitative determination of the HEVNP biodistribution in mice. Overall, our results indicate that the developed [^68^Ga]Ga-DOTA-HEVNP is a promising platform for targeted delivery of therapeutics and diagnostics to hepatocytes.

## Supporting Information Available

Additional experimental details and materials used in cell culture and uptake; all the % ID/g values of [^68^Ga]Ga-DOTA-HEVNPs after the *ex vivo* study in mice; and graphical representation of the membrane-bound fractions of the [^68^Ga]Ga-DOTA-HEVNP and free [^68^Ga]GaCl_3_ on hepatocytes and macrophages (PDF)
