# Supplementary material for: Development of 68Ga-Labeled Hepatitis E Virus Nanoparticles for Targeted Drug Delivery and Diagnostics with PET
Source: Mol Pharm. 2022 Jul 20;19(8):2971–9. doi: 10.1021/acs.molpharmaceut.2c00359 (PMC9346612; doi:10.1021/acs.molpharmaceut.2c00359)
Supplement: Supplementary file 1 — mp2c00359_si_001.pdf [file mp2c00359_si_001.pdf]

Supplementary Information

Development of ^{68}Ga-labeled Hepatitis E virus nanoparticles for targeted drug delivery and diagnostics with PET

Elisavet Lambidis[a], Chun-Chieh Chen[b], Mo Baikoghli[b], Surachet Imlimthan[a], You Cheng Khng[a], Mirkka Sarparanta[a], R. Holland Cheng*[,b], Anu J. Airaksinen*[,a,c]

[a]Department of Chemistry, Radiochemistry, University of Helsinki, Helsinki, FI-00014, Finland
[b]Department of Molecular and Cellular Biology, University of California, Davis, CA 95616, U.S.A.
[c]Turku PET Centre, Department of Chemistry, University of Turku, Turku, FI-20520, Finland

Experimental Procedures

All chemicals and solvents were obtained from commercial providers and they were used without further purification.

Cell culture:

Cell culturing flasks and well plates were obtained from Corning Inc. (Corning, NY, U.S.A.). McCoy's 5A (for HCT 116 cells) and Dulbecco's Modified Eagle Medium (RAW 264.7 cells) cell culture media were purchased from ATCC (Manassas, VA, U.S.A.) and Corning, respectively. DMEM (for Hep G2), CO_2-independent cell medium, GlutaMAX (1×), sodium pyruvate (100 mM), fetal bovine serum (FBS), Penicillin-Streptomycin (10,000 U/ml), non-essential amino acids (100×NEAA), Dulbecco's phosphate buffer saline (10×DPBS), TrypLE™ Express (1×), and sterile vacuum filter/storage bottle unit (250–500 ml, 0.22 µm PES membrane) were purchased from Life Technologies Gibco (Carlsbad, CA, U.S.A.). The Heracell VIOS 160i incubator (Thermo Fisher Scientific, Waltham, MA, U.S.A.) was maintained at 37 °C, 5% CO_2, and 95% relative humidity.

Cell culture media preparation:

The cell culture media were supplemented with 10% FBS, 1% GlutaMAX, 1% sodium pyruvate and 1% Penicillin-Streptomycin, and aseptically filtered through sterile 0.22-µm filter before use. In addition to this general procedure, DMEM medium for Hep G2 cell line was further supplemented with 1% NEAA.

Cell studies of [^{68}Ga]Ga-DOTA-HEVNPs and controls in hepatocytes, macrophages and colorectal cancer cells:

The cell medium was removed and collected into a scintillation tube at the designed time. *Free fraction collection*: the cells were washed with 1 mL of ice-cold 0.01 M PBS (pH 7.4) and the PBS was collected in the same tube. *Membrane-bound fraction collection*: the addition of 1 mL of ice-cold glycine buffer (0.05 M, pH 2.8). The cells were incubated for 5 min on ice. The buffer was then collected in a new scintillation tube. This procedure was repeated once and the buffer was collected in the same tube. The treated cells were washed with 1 mL of ice-cold 0.01 M PBS (pH 7.4) and this was then collected in the same tube. *Internalized fraction*

collection: 1 mL of 1 M NaOH was added and the cells were incubated for 10 min at RT. NaOH was collected in a new scintillation tube. The cells were washed twice with 1 mL of ice-cold 0.01 M PBS and the buffer was collected both times in the same tube. The radioactivity was detected in each tube using the Wizard automated gamma-counter. The percent internalization was normalized with total added activity and calculated as: %Internalization = 100 × CPM of internalized fraction/SUM of the CPM in all fractions. The measurements were done in triplicate for every time point and sample under study.

Results

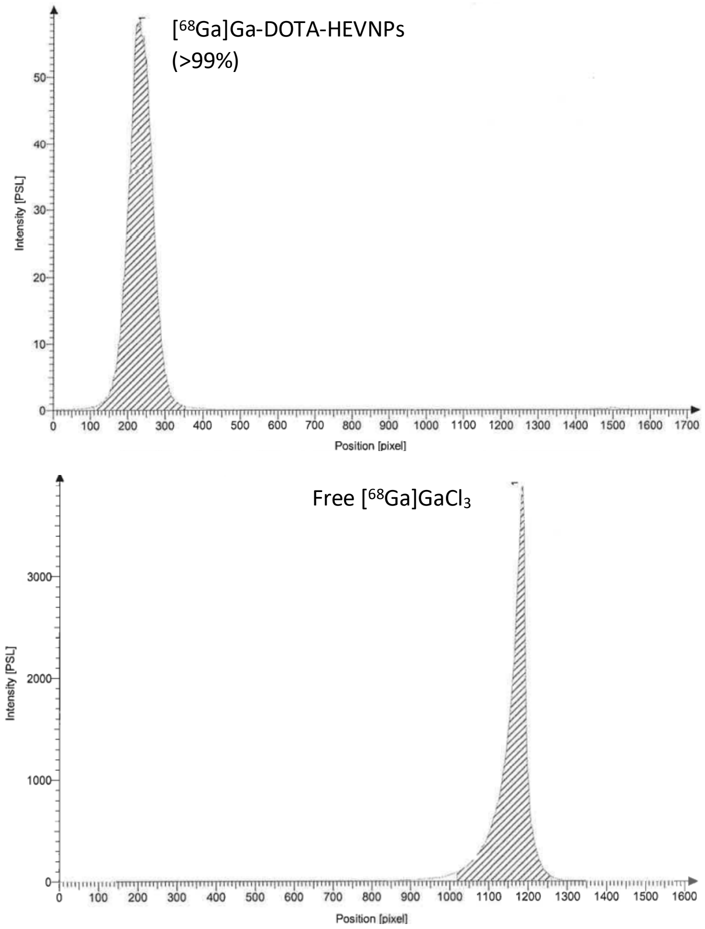

Fig.S1: Radio-TLC chromatograms of pure [^{68}Ga]Ga-DOTA-HEVNPs (top) and free ^{68}Ga-radionuclide (bottom); conditions: Whatman 1 paper chromatography with 0.5 mM DTPA.

Table S1: The average %ID/g values obtained from the *ex vivo* biodistribution of the [^{68}Ga]Ga-DOTA-HEVNPs tracer in healthy mice (n = 3-5). S.D.: standard deviation.

**Tissue**	**15 min**		**30 min**		**60 min**		**120 min**	
	**%ID/g**	**S.D.**	**%ID/g**	**S.D.**	**%ID/g**	**S.D.**	**%ID/g**	**S.D.**
**Urine**	97.34	83.66	222.49	149.24	142.61	98.55	46.45	55.48
**Blood**	3.9	1.47	3.42	0.7	2.87	2.32	2.11	0.67
**Pancreas**	0.52	0.22	0.6	0.08	0.49	0.43	0.48	0.18
**Spleen**	12.11	2.28	12.14	3.53	17.21	7.7	15.05	3.44
**Kidney**	2.12	0.26	1.63	0.25	1.48	1.03	1.09	0.17
**Gallbladder**	11.51	11.23	21.53	12.8	17.4	10.58	15.16	8.8
**Liver**	55.04	13.83	69.28	10.86	96.34	58.65	62.82	11.04
**Lung**	3.99	1.64	4.09	1.09	3.42	2.94	2.49	0.77
**Heart**	1.77	0.75	1.59	0.32	1.24	1.01	1.06	0.37
**Muscle**	0.46	0.21	0.6	0.19	0.42	0.42	0.44	0.19
**Occipital bone**	1.29	0.85	1.32	0.84	1.44	1.09	1.62	0.8
**Bone with marrow**	2.18	0.89	2.3	0.87	3.42	3.1	3.2	0.99
**Brain**	0.16	0.1	0.16	0.08	0.13	0.12	0.13	0.06
**Stomach**	0.25	0.12	0.23	0.09	0.3	0.33	0.23	0.09
**Small intestine**	0.4	0.06	0.46	0.13	0.6	0.49	0.6	0.27
**Large intestine**	0.23	0.07	0.28	0.09	0.28	0.24	0.48	0.16
**Skin**	1.06	0.53	1.11	0.54	0.86	0.76	1.08	0.3

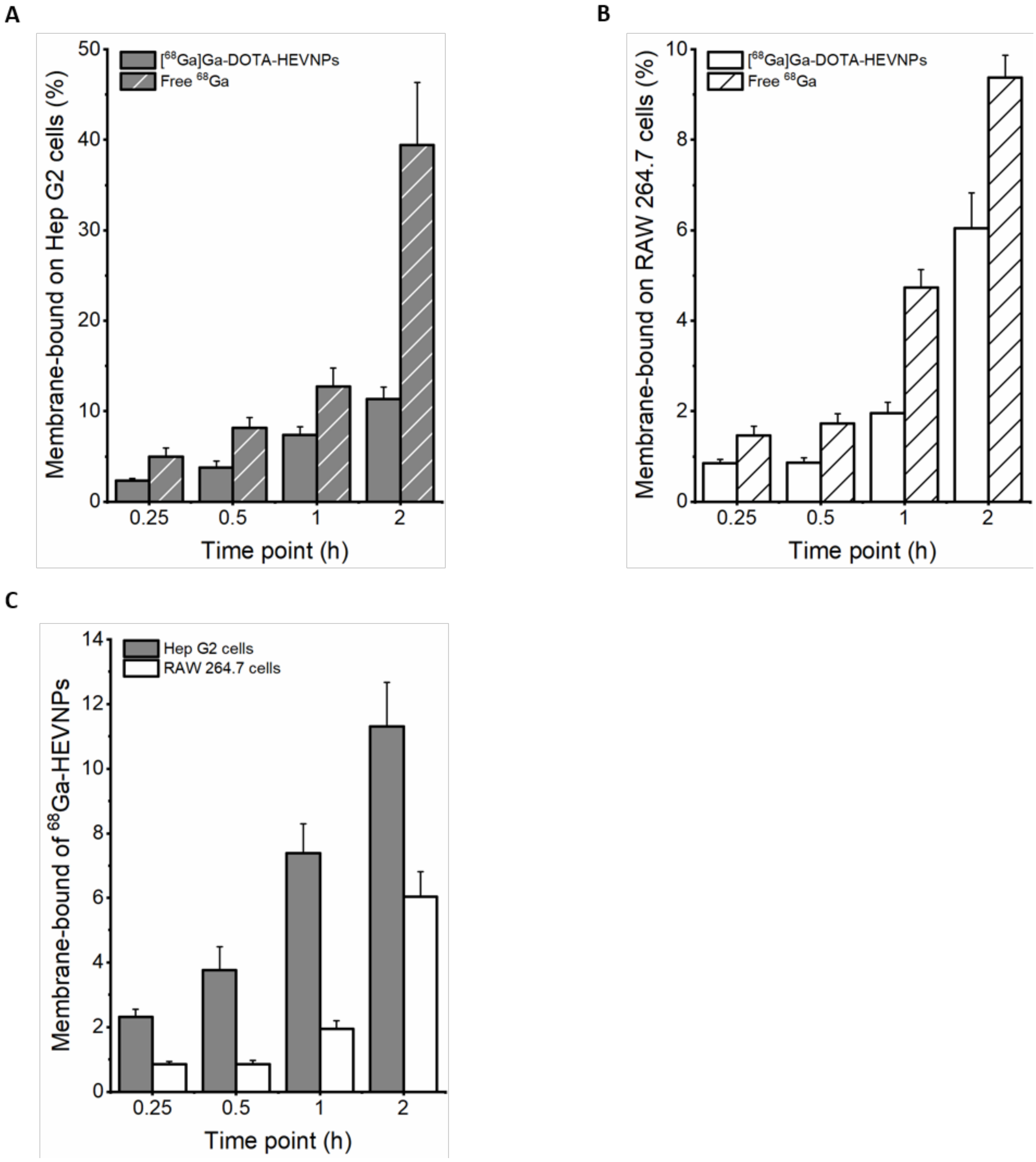

Fig.S2: Membrane-bound fractions of [^{68}Ga]Ga-DOTA-HEVNPs and [^{68}Ga]$GaCl_3$ in (A) hepatocytes (Hep G2) and (B) macrophages (RAW 264.7). The comparison between the two cell lines is shown for [^{68}Ga]Ga-DOTA-HEVNPs (C). The cell uptake assay was done at 37 ^{o}C up to 2 h after the initiation of the incubation. The columns represent the average ± standard deviation (n = 3).
